# CSP and articles in Social Sciences and Humanities in Health: what has been, what is to come

**DOI:** 10.1590/0102-311XEN107424

**Published:** 2024-07-22

**Authors:** Suely Deslandes

**Affiliations:** 1 Instituto Nacional de Saúde da Mulher, da Criança e do Adolescente Fernandes Figueira, Fundação Oswaldo Cruz, Rio de Janeiro, Brasil.

*Cadernos de Saúde Pública* (CSP) celebrates its 40th anniversary, keeping alive its mission to broadly publish in all disciplinary areas of Public Health. But how have the Social Sciences and Humanities in Health participated in this trajectory? To discussion this, we analyzed the Social Sciences and Humanities in Health production published by CSP in the last 20 years (2004-2023).

This time interval contains 284 articles that were analyzed and approved by the associate editors of Social Sciences. The share of Social Sciences and Humanities in Health texts in the total number of articles published by the journal has been very modest in the last 20 years, ranging from about 2% (2008) to 9% (2013). Some hypotheses may enliven our discussion, such as the existence of other excellent journals with a profile better dedicated to Social Sciences and Humanities in Health publications that attract a greater number of such articles and the “flight of articles” to international journals. However, since about 80%-90% of submitted texts are rejected, the issue of article quality requires addressal as the evaluation system of the editors-in-chief or subsequent peer reviews deemed these texts as having theoretical or methodological problems or failing to offer original reflections. This points to the lack of training in Social Sciences and Humanities in Health (and especially Public Health), reaffirming the need for greater investments in a field that is interdisciplinary by definition and, therefore, with many researchers from different scientific areas, demanding theoretical-methodological-based learning. Studies have been pointed out the low or irregular offer of courses in methodologies in Social Sciences and social theory for students of master’s and PhD programs for some time ^1^ ^,^ ^2^ ^,^ ^3^. The decreasing number of Social Sciences and Humanities in Health professors in postgraduate programs in Public Health ^4^ deserves analysis and hinders the task of training, affecting the quality of scientific productions in general and that of articles.

Furthermore, analysis of the set of Social Sciences articles published by CSP can unhesitantly state that it represents the enormous thematic diversity and the most relevant debates in the field.

Studies on health care policies, programs, services, and aspects constituted the main published thematic set, indicating that the historical involvement of Social Sciences and Humanities in Health as a field of knowledge linked to critical analysis and improvement of the Brazilian Unified National Health System and public health policies remains alive. Next, analysis highlighted articles on women’s sexual and reproductive rights. However, its gradually consolidated the visibility of other subjects, such as studies on men and different masculinities, trans people, and issues related to the health of LGBTQI people. This context significantly featured the issue of abortion, denouncing the numerous difficulties and prejudices women suffer when resorting to the legal interruption of their pregnancies and the barriers to implementing public policies that recognize the right of women to decide having children. The different forms of violence and their impacts on health were the third most popular thematic group, especially in texts that address violence against cis and trans women, forms of care, and relationships with health agents. Themes that are equally dear to the field of Social Sciences and Humanities in Health were also highlighted, such as the representations and practices related to the health-disease-care process, workers’ health and work process, sexualities and STI-HIV, and mental health.

In addition to this general thematic framework these lines described in very superficial lines, we highlight two valuable contributions in this set published by CSP: discussions on qualitative methodological procedures and those of social theory. These articles reflect on social thinking in health in Latin America, the relevance of social determination, conceptions of risk, and address the contribution of seminal authors such as Pierre Bourdieu, Roger Bastide, among others. Others studies address theoretical-practical themes of methodological operationalization and, together with theoretical research texts, are essential to training researchers in our field.

In the painful context of the COVID-19 emergency, the Social Sciences and Humanities in Health articles published by CSP discussed topics of vital relevance to understanding how Brazil experienced the pandemic. Thus, articles in the period discussed the undeniable marks of structural racism exposed by deaths to COVID-19, the media representations of the pandemic, the debate on vaccines on social media, the dimensions of care in an unprecedented context in recent history. A research agenda for Social Sciences and Humanities in Health in times of COVID-19 was also the subject of an editorial in 2021.

CSP also supported and gave visibility to the Congresses of Social Sciences and Humanities in Health. At each edition of this seminal congress, it published an editorial, and its organizers could disseminate the main points of the debate and the political agendas put forward by this community of research and practices.

In summary, CSP has contributed with relevant articles in Social Sciences and Humanities in Health, continuously supporting the production and initiatives in the field. However, this participation could be greater, guaranteeing both the space for established productions with the utmost relevance to Public Health and giving visibility to the many studies that address the necessary epistemic, theoretical, research practices, and service transformations and ruptures: discussions that emerge in the context of decolonialities, identity situationalities, conditions that signal oppression and denial of rights, empowerment and agency, the transformations processed by digital capitalism and its technologies, environmental justice and ecological crisis, among many other themes that require us to reflect on the “present time” and that can enrich the community of debates represented by CSP.

That is the invitation, that is the challenge. Let CSP establish the living space of the restless, creative, and transformative debate that characterizes reflections on Social Sciences and Humanities in Health!
