# Supplementary material for: CSP and articles in Social Sciences and Humanities in Health: what has been, what is to come
Source: Cad Saude Publica. 2024 Jul 22;40(7):e00107424. doi: 10.1590/0102-311XEN107424 (PMC11286263; doi:10.1590/0102-311XEN107424)
Supplement: Translation

# Translation: CSP e os artigos de Ciências Sociais e Humanas em Saúde: o que tem sido, o que está por vir

**DOI:** 10.1590/0102-311XPT107424

**Authors:** Suely Deslandes

**Affiliations:** 1 Instituto Nacional de Saúde da Mulher, da Criança e do Adolescente Fernandes Figueira, Fundação Oswaldo Cruz, Rio de Janeiro, Brasil.

*Cadernos de Saúde Pública* (CSP) comemora 40 anos mantendo viva a missão de publicação ampla em todos as áreas disciplinares da Saúde Coletiva. Mas como tem sido a participação das Ciências Sociais e Humanas em Saúde nessa trajetória? Para realizar essa discussão, analisamos a produção de Ciências Sociais e Humanas em Saúde publicada em CSP nos últimos 20 anos (2004-2023).

Nesse intervalo temporal temos um total de 284 artigos que foram analisados e aprovados pelos editores associados de Ciências Sociais. Podemos mesmo dizer que nos últimos 20 anos a participação dos textos de Ciências Sociais e Humanas em Saúde no total de artigos publicados pela revista foi muito modesta, variando de cerca de 2% (2008) a 9% (2013). Algumas hipóteses podem animar nossa discussão, como a existência de outras excelentes revistas com perfil mais dedicado à publicação em Ciências Sociais e Humanas em Saúde, que atraem um número maior de artigos da área, e a “fuga de artigos” para revistas internacionais. Todavia, com uma taxa de cerca de 80% a 90% de textos recusados dentre os submetidos, a questão da qualidade dos artigos é um tema que precisa ser enfrentado. São textos que no sistema de avaliação das editoras-chefes ou, posteriormente, na análise por pares foram apontados como apresentando problemas de ordem teórica, metodológica ou não aportando reflexões originais. Tal quadro alerta para a questão da formação de Ciências Sociais e Humanas em Saúde no campo da Saúde e da Saúde Coletiva em particular, reafirmando a necessidade de maiores investimentos em um campo que é interdisciplinar por definição e, portanto, com muitos pesquisadores oriundos de distintas áreas científicas que demandam aprendizado de base teórico-metodológica. A baixa ou irregular oferta de cursos de metodologias em Ciências Sociais e de teoria social para alunos de programas de mestrado e doutorado tem sido apontada em estudos há algum tempo ^1^ ^,^ ^2^ ^,^ ^3^. O decrescente número de professores da área de Ciências Sociais e Humanas em Saúde nos programas de pós-graduação em Saúde Coletiva ^4^ é um fato que merece ser analisado e torna ainda mais difícil a tarefa de formação, repercutindo na qualidade das produções científicas em geral e também nos artigos.

Outrossim, ao analisar o conjunto dos artigos de Ciências Sociais publicados em CSP podemos afirmar sem qualquer hesitação que está ali representada a enorme diversidade temática e os debates mais relevantes do campo.

Estudos dedicados à análise de políticas, programas, serviços e aspectos dos atendimentos em saúde foram o principal conjunto temático publicado, indicando que o histórico envolvimento das Ciências Sociais e Humanas em Saúde como campo de conhecimento vinculado à análise crítica e aperfeiçoamento do Sistema Único de Saúde (SUS) e das políticas públicas de saúde se mantém vivo. A seguir, artigos que tratam dos direitos sexuais e reprodutivos das mulheres foram destaque, mas gradativamente se consolida a visibilidade de outros sujeitos, como os textos que colocam em foco os homens e as distintas masculinidades, as pessoas trans e questões relacionadas à saúde de pessoas LGBTQI. Nesse conjunto, o tema do aborto foi significativamente presente, denunciando as inúmeras dificuldades e preconceitos que mulheres sofrem ao recorrer à interrupção legal da gravidez, bem como as barreiras para a implantação de políticas públicas que reconheçam o direito de mulheres decidirem por ter ou não filhos. As diferentes violências e seus impactos na saúde foram o terceiro conjunto temático que reuniu mais artigos, especialmente em textos que tratam da violência contra mulheres cis e trans, as formas de atendimento e as relações com os agentes do campo da Saúde. Também se destacaram os temas igualmente caros ao campo das Ciências Sociais e Humanas em Saúde, como as representações e práticas relacionadas ao processo saúde-doença-atenção, processo de trabalho e a saúde dos trabalhadores, sexualidades e IST-HIV e saúde mental.

Além desse quadro temático geral, aqui descrito em linhas muito superficiais, destacamos duas valiosas contribuições em textos também publicados em CSP: os estudos que discutem procedimentos metodológicos qualitativos e os de teoria social. São artigos que refletem sobre o pensamento social em saúde na América Latina, a atualidade da determinação social, das concepções de risco, além de abordar a contribuição de autores seminais como Pierre Bourdieu, Roger Bastide, entre outros. Há também os trabalhos que tratam temas teórico-práticos da operacionalização metodológica e, juntamente com os textos de pesquisa teórica, se mostram essenciais à formação de pesquisadores de nosso campo.

No doloroso marco da emergência da COVID-19, os artigos de Ciências Sociais e Humanas em Saúde publicados em CSP discutiram temas de vital relevância para a compreensão de como a pandemia foi vivida no Brasil. Assim, as inegáveis marcas do racismo estrutural expostas no cenário das mortes por COVID-19, as representações midiáticas da pandemia, o debate sobre vacinas nas redes sociais, as dimensões do cuidado em um contexto sem precedentes na história recente foram assuntos tratados em artigos nesse período. Uma agenda de pesquisa para as Ciências Sociais e Humanas em Saúde em tempo de COVID-19 também foi tema de editorial em 2021.

CSP também se fez presente no apoio e visibilização dos Congressos de Ciências Sociais e Humanas em Saúde. A cada edição desse congresso seminal do campo, um editorial foi publicado e seus organizadores puderam ali divulgar os principais pontos do debate e das agendas políticas colocadas por essa comunidade de pesquisa e práticas.

Em síntese, poderíamos dizer que CSP tem contribuído com artigos relevantes em Ciências Sociais e Humanas em Saúde e que segue apoiando a produção e as inciativas do campo, mas essa participação pode ser ainda maior, garantindo tanto os espaços para as produções consagradas e de máxima relevância para a Saúde Coletiva, como dando visibilidade aos muitos trabalhos que tratam das necessárias transformações e rupturas, sejam epistêmicas, teóricas e das práticas de pesquisa e dos serviços. Discussões que emergem no contexto das decolonialidades, das situcionalidades identitárias, das condições que sinalizam opressões e negação de direitos, mas também empoderamento e agência, das transformações processadas pelo capitalismo digital e suas tecnologias, da justiça ambiental e crise ecológica, entre tantos outros temas que nos exigem reflexão sobre o “tempo presente” e que podem enriquecer a comunidade de debates que CSP representa.

Eis o convite, eis o desafio. Façam de CSP o espaço vivo do debate inquieto, criativo e transformador que caracteriza a reflexão das Ciências Sociais e Humanas em Saúde!

# Translation: CSP y los artículos de Ciencias Sociales y Humanas en Salud: lo que ha sido, lo que está por venir

**DOI:** 10.1590/0102-311XES107424

**Authors:** Suely Deslandes

**Affiliations:** 1 Instituto Nacional de Saúde da Mulher, da Criança e do Adolescente Fernandes Figueira, Fundação Oswaldo Cruz, Rio de Janeiro, Brasil.

*Cadernos de Saúde Pública* (CSP) celebra 40 años de mantener viva su misión de publicación amplia en todas las áreas disciplinarias de la Salud Colectiva. Pero ¿cómo ha sido la participación de las Ciencias Sociales y Humanas en Salud en esta trayectoria? Para llevar a cabo esta discusión, analizamos la producción de Ciencias Sociales y Humanas en Salud publicada en CSP en los últimos 20 años (2004-2023).

En este intervalo temporal, tenemos un total de 284 artículos que fueron analizados y aprobados por los editores asociados de Ciencias Sociales. Incluso podemos decir que en los últimos 20 años la participación de los textos de Ciencias Sociales y Humanas en Salud en el número total de artículos publicados por el periódico ha sido muy modesta, variando entre alrededor del 2% (2008) al 9% (2013). Algunas hipótesis pueden animar nuestra discusión, como la existencia de otras revistas excelentes con un perfil más dedicado a la publicación en Ciencias Sociales y Humanas en Salud que atraen un mayor número de artículos en el área y la “fuga de artículos” hacia revistas internacionales. Sin embargo, con una tasa de alrededor del 80% al 90% de textos rechazados entre los enviados, la cuestión de la calidad de los artículos es un aspecto que se debe enfrentar. Se trata de textos que en el sistema de evaluación de las editoras jefes o, posteriormente, en el análisis de pares fueron identificados como textos que presentan problemas teóricos o metodológicos o que no aportan reflexiones originales. Esta situación alerta sobre la cuestión de la formación de la Ciencias Sociales y Humanas en Salud en el ámbito de la Salud y de la Salud Colectiva en particular, reafirmando la necesidad de una mayor inversión en un campo que es interdisciplinar por definición y, por lo tanto, con muchos investigadores provenientes de diferentes áreas científicas que demandan aprendizaje de base teórico-metodológica. La baja o irregular oferta de cursos de metodologías en Ciencias Sociales y de teoría social para estudiantes de programas de máster y doctorado ha sido señalada en estudios desde hace un tiempo ^1^ ^,^ ^2^ ^,^ ^3^. El decreciente número de profesores del área de Ciencias Sociales y Humanas en Salud en los programas de posgrado en Salud Colectiva ^4^ es un hecho que merece ser analizado y que dificulta aún más la tarea formativa, repercutiendo en la calidad de las producciones científicas en general y también en los artículos.

Además, al analizar el conjunto de los artículos de Ciencias Sociales publicados en CSP, podemos afirmar sin dudar que allí están representados la enorme diversidad temática y los debates más relevantes de este campo.

Los estudios dedicados al análisis de políticas, programas, servicios y aspectos de la atención a la salud fueron el principal conjunto temático publicado, lo que indica que la participación histórica de las Ciencias Sociales y Humanas en Salud como campo de conocimiento vinculado al análisis crítico y al perfeccionamiento del Sistema Único de Salud brasileño y de las políticas públicas de salud se mantiene viva. A continuación, se destacaron artículos que abordan los derechos sexuales y reproductivos de las mujeres, pero gradualmente se consolida la visibilidad de otros sujetos, como los textos que se centran en los hombres y en las distintas masculinidades, las personas trans y cuestiones relacionadas a la salud de las personas LGBTQI. En este conjunto, el tema del aborto estuvo significativamente presente, denunciando las innumerables dificultades y prejuicios que sufren las mujeres al recurrir a la interrupción legal del embarazo, así como las barreras para la implementación de políticas públicas que reconozcan el derecho de las mujeres a decidir si tener o no hijos. Las diferentes violencias y sus impactos en la salud fueron el tercer conjunto temático que reunió más artículos, especialmente en textos que abordan la violencia contra las mujeres cis y trans, las formas de atención y las relaciones con los agentes del ámbito sanitario. También se resaltaron temas igualmente importantes para el campo de las Ciencias Sociales y Humanas en Salud, como las representaciones y prácticas relacionadas con el proceso de salud-enfermedad-atención, el proceso de trabajo y la salud de los trabajadores, sexualidades e ITS-VIH y la salud mental.

Además de este marco temático general, descrito aquí en términos muy superficiales, destacamos dos contribuciones valiosas en textos también publicados en CSP: los estudios que discuten procedimientos metodológicos cualitativos y los de teoría social. Se trata de artículos que reflexionan sobre el pensamiento social en salud en América Latina, el estado actual de la determinación social, concepciones de riesgo, además de abordar los aportes de autores seminales como Pierre Bourdieu, Roger Bastide, entre otros. También existen trabajos que abordan temas teórico-prácticos de operativización metodológica y, junto con textos de investigación teórica, son fundamentales para la formación de investigadores en nuestro campo.

En el doloroso hito de la emergencia de la COVID-19, los artículos de Ciencias Sociales y Humanas en Salud publicados en CSP discutieron temas de vital relevancia para comprender cómo se vivió la pandemia en Brasil. Así, las innegables marcas del racismo estructural expuestas en el escenario de las muertes por COVID-19, las representaciones mediáticas de la pandemia, el debate sobre las vacunas en las redes sociales, las dimensiones del cuidado en un contexto sin precedentes en la historia reciente fueron temas abordados en los artículos de este período. Una agenda de investigación para las Ciencias Sociales y Humanas en Salud en tiempos de COVID-19 también fue tema de un editorial en el 2021.

CSP también estuvo presente en el apoyo y visibilización de los Congresos de Ciencias Sociales y Humanas en Salud. En cada edición de este proficuo congreso del campo, se publicó un editorial, y sus organizadores pudieron dar a conocer los principales puntos del debate y de las agendas políticas planteadas por esta comunidad de investigación y prácticas.

En resumen, podríamos decir que CSP ha aportado artículos relevantes en Ciencias Sociales y Humanas en Salud y que siguen apoyando la producción y las iniciativas del campo, pero esta participación podría ser aún mayor, garantizando tanto espacios para las producciones reconocidas y de máxima relevancia para la Salud Colectiva como dando visibilidad a los múltiples trabajos que abordan las transformaciones y rupturas necesarias, ya sean epistémicas, teóricas, y de las prácticas de investigación y de los servicios. Discusiones que emergen en el contexto de descolonialidades, de las situaciones identitarias, de las condiciones que señalan opresión y negación de derechos, pero también empoderamiento y agencia, de las transformaciones procesadas por el capitalismo digital y sus tecnologías, de la justicia ambiental y crisis ecológica, entre tantos otros temas que nos exigen reflexionar sobre el “tiempo presente” y que pueden enriquecer la comunidad de debates que CSP representa.

Aquí está la invitación, aquí está el desafío. ¡Hacer de CSP el espacio vivo para el debate inquieto, creativo y transformador que caracteriza la reflexión de las Ciencias Sociales y Humanas en Salud!
